# The Buffalo General Hospital

**Published:** 1883-08

**Authors:** 


### The Buffalo General Hospital.

We learn that at a recent meeting of the medical and surgical staff of the hospital, the subject was discussed of filling the position on the active surgical staff, vacated by Dr. Miner on account of continued ill health. The name of Dr. Park, the newly elected lecturer at the medical college, was mentioned for that position, and objections being raised, the question was deferred to another meeting. It occurs to us to inquire, why should the Buffalo General Hospital follow the example of the college and import a surgeon from a neighboring city for a position on its staff? We do not question the right of the college to ignore the professional talent of this city, and seek its instructors from abroad. Such action concerns only the small coterie of medical men who comprise its faculty. The General Hospital, however, is placed upon a very different basis. It needs for its maintenance the sympathy and co-operation of the medical profession of this city, and it can not afford to ignore the very respectable surgical talent which the profession contains and seek a stranger upon whom to confer its honors, simply because the college desires it with a view to utilize its patients for clinical instruction for its classes. The hospital should be something more than an appendage to the college. Its name warrants the opinion that it should hold an independent position open to the wants of all the profession, even as it aims to recognize the rights of all, especially of our city surgeons.

An important question for the hospital is that of support, and without an adequate endowment, such support depends largely upon the medical profession. How can it be expected that the profession will favor the hospital if the trustees continue to disregard the laudable ambition of its members by conferring appointments upon men whose only recommendation for its honors consists in their relations with the college?

This subject has been freely discussed in professional circles, and much ill feeling engendered towards the hospital for appointments which have been made on its staff, not for professional merit and attainments, but from social and college influences; until it has come to be a saying, that the college runs the hospital, and makes use of it to further its own selfish purposes.

We cannot say that all the present medical and surgical staff are representative men. Men of wider attainments and a higher order of ability have failed to seek recognition because of the uselessness of such an effort on account of the predominance of college influence in the board of trustees of the hospital.

The time is opportune for the hospital to recognize the true status of feeling prevailing in the profession of the city. A complete revolution in its medical and surgical staff would bring the institution more in sympathy with our best medical men, and through them with a wider popular constituency, while such a change would improve the mortuary statistics, which for many years have not been creditable to that institution. Such a revolution would satisfy the medical profession of the city and bring it in more cordial relations than now exist.

